# Prognostic factors and survival in MEN1 patients with gastrinomas: Results from the DutchMEN study group (DMSG)

**DOI:** 10.1002/jso.25667

**Published:** 2019-08-10

**Authors:** Dirk‐Jan van Beek, Sjoerd Nell, Carolina R.C. Pieterman, Wouter W. de Herder, Annenienke C. van de Ven, Olaf M. Dekkers, Anouk N. van der Horst‐Schrivers, Madeleine L. Drent, Peter H. Bisschop, Bas Havekes, Inne H. M. Borel Rinkes, Menno R. Vriens, Gerlof D. Valk

**Affiliations:** ^1^ Department of Endocrine Surgical Oncology University Medical Center Utrecht Utrecht The Netherlands; ^2^ Department of Endocrine Oncology University Medical Center Utrecht Utrecht The Netherlands; ^3^ Department of Internal Medicine Erasmus Medical Center Rotterdam The Netherlands; ^4^ Department of Endocrinology Radboud University Medical Center Nijmegen The Netherlands; ^5^ Departments of Endocrinology and Metabolism and Clinical Epidemiology Leiden University Medical Center Leiden The Netherlands; ^6^ Department of Endocrinology, University Medical Center Groningen University of Groningen Groningen The Netherlands; ^7^ Department of Internal Medicine, Section of Endocrinology, Amsterdam UMC location VU University Medical Center Amsterdam The Netherlands; ^8^ Department of Endocrinology and Metabolism Amsterdam UMC location Academic Medical Center Amsterdam The Netherlands; ^9^ Department of Internal Medicine, Division of Endocrinology Maastricht University Medical Center Maastricht The Netherlands

**Keywords:** multiple endocrine neoplasia type 1, neuroendocrine tumor, oncology, Zollinger‐Ellison syndrome

## Abstract

**Background and objectives:**

Gastrinomas are the most prevalent functioning neuroendocrine tumors (NET) in multiple endocrine neoplasia type 1 (MEN1). Guidelines suggest medical therapy in most patients, but surgery may be considered in a subgroup. Currently, factors to guide management are necessary. This population‐based cohort study assessed prognostic factors of survival in patients with MEN1‐related gastrinomas.

**Methods:**

Patients with MEN1 having gastrinomas were identified in the Dutch MEN1 database from 1990 to 2014 based on fasting serum gastrin (FSG) levels and/or pathology. Predictors of overall survival were assessed using Cox regression.

**Results:**

Sixty‐three patients with gastrinoma (16% of the MEN1 population) were identified. Five‐ and 10‐year overall survival rates were 83% and 65%, respectively. Prognostic factors associated with overall survival were initial FSG levels ≥20x upper limit of normal (ULN) (hazard ratio [HR], 6.2 [95% confidence interval, 1.7‐23.0]), pancreatic NET ≥2 cm (HR 4.5; [1.5‐13.1]), synchronous liver metastases (HR 8.9; [2.1‐36.7]), gastroduodenoscopy suspicious for gastric NETs (HR 12.7; [1.4‐115.6]), and multiple concurrent NETs (HR 5.9; [1.2‐27.7]).

**Conclusion:**

Life expectancy of patients with MEN1 gastrinoma is reduced. FSG levels and pancreatic NETs ≥2 cm are prognostic factors. FSG levels might guide surveillance intensity, step‐up to additional diagnostics, or provide arguments in selecting patients who might benefit from surgery.

**Abbreviations**
CI: Confidence interval
CT: Computed tomography
DMSG: Dutch MEN Study Group
dpNET: Duodenopancreatic neuroendocrine tumor
ENETS: European Neuroendocrine Tumor Society
EUS: Endoscopic ultrasonography
FSG: Fasting serum gastrin
HR: Hazard ratio
MEN1: Multiple endocrine neoplasia type 1
MRI: Magnetic resonance imaging
NET: Neuroendocrine tumor
NF‐pNET: Nonfunctioning pancreatic neuroendocrine tumor
OS: Overall survival
pHPT: Primary hyperparathyroidism
pNET: Pancreatic neuroendocrine tumor
PPI: Proton pump inhibitor
SD: Standard deviation
ULN: Upper limit of the normal of the reference range
UMC: University Medical Center

## 1 INTRODUCTION

Multiple endocrine neoplasia type 1 (MEN1) is a rare autosomal dominant disorder caused by a mutation in the *MEN1* gene leading to a combination of endocrine and nonendocrine tumors.^1^ Duodenopancreatic neuroendocrine tumors (dpNETs) are a common manifestation and have a prevalence of 56% in the Dutch MEN1 population.^2^ Gastrinomas are the most frequently encountered functioning dpNETs and occur in approximately 30% of patients with MEN1.^3^ These tumors produce gastrin which induces gastric acid hypersecretion and subsequently leads to ulcerative peptic disease and gastrointestinal bleeding, known as the Zollinger‐Ellison syndrome.^4^ MEN1‐related gastrinomas are generally located in the duodenal submucosa and are rarely found in the pancreas.^5^, ^6^ Duodenal gastrinomas are often small (<1 cm), multiple and accompanied by pancreatic neuroendocrine tumors (pNETs).^6^ Approximately 70% to 80% of surgically treated patients have lymph node metastases, and 10% present with synchronous liver metastases.^7^, ^8^

Gastric acid hypersecretion‐related complications used to be the leading cause of death in patients with MEN1 having gastrinomas before the widespread use of proton pump inhibitors (PPIs).^9^, ^10^ Nowadays, compared with the general population, patients with MEN1 have a seriously decreased life expectancy mainly caused by malignant dpNETs.^2^ However, the reported prognosis of patients with MEN1 gastrinoma varies widely.^8^, ^11^, ^12^, ^13^ In the French cohort, studied from 1956 to 2005, gastrinomas have been reported as an independent risk factor for death.^9^ Actual data on MEN1 gastrinoma survival are scarce and survival rates are difficult to interpret since patients are diagnosed and treated differently among studies. In addition, the understanding that MEN1 gastrinomas mostly originate in the duodenum instead of the formerly assumed pancreatic origin, emphasizes the need for new studies. Besides, data regarding the long‐term natural history are important, because guidelines suggest symptomatic management using PPIs in the majority of patients.^14^, ^15^ Nevertheless, the only potentially curative oncological treatment remains surgery. Pancreaticoduodenectomy offers the possibility to achieve a biochemical cure for MEN1‐related duodenal gastrinomas.^8^ However, controversies exist regarding the timing and the extent of surgery, considering the unpredictable tumor course and morbidity associated with extensive surgery.^14^, ^15^ Therefore, the necessity of prognostic factors to guide therapy has recently been underscored.^16^ Since gastrinomas are hormone producing tumors, we hypothesized that, besides known dpNET‐related prognostic factors such as pNET size and liver metastases, gastrin levels might predict survival in this population.^17^ Therefore the present study aims to assess prognostic factors and survival in patients with MEN1 having gastrinomas.

## 2 MATERIALS AND METHODS

### 2.1 Study design

Patients were selected from the national Dutch MEN1 database from the DutchMEN Study Group (DMSG).^18^ Patients with MEN1 aged 16 years and older and under treatment in one of the eight University Medical Centers (UMCs) are included. In each center, patients were identified by reviewing hospital databases of medical conditions and diseases. MEN1 diagnosis was established according to the guidelines.^14^ Over 90% of the Dutch MEN1 population is included. Clinical and demographic data were collected longitudinally every quarter from 1990 to 2014 by standardized medical record review, according to a predefined protocol. The protocol was approved by the Medical Ethics Committees of all UMCs.

### 2.2 Patient selection

The diagnosis of gastrinomas in patients with MEN1 was challenging, since the reference standard, provocative tests using secretin, is not widely available and routine measurements of basal acid output at gastroduodenoscopy were not routinely performed. Therefore, based on stringent criteria we aimed to identify those patients of whom we were confident of having gastrinomas, also using subsequently elevated fasting serum gastrin (FSG) levels. Gastrinoma diagnosis was based on (a) pathology reports of gastrin immunohistochemistry positive tumors or (b) elevated FSG levels, or (c) gastroduodenoscopy suspicious for gastrinoma, or a combination of these. Serum gastrin reference values were obtained from all UMCs over the study period. FSG levels were calculated as a factor of the upper limit of normal (ULN) of the reference values. Gastrinoma diagnosis was considered certain when FSG levels were increased more than a 10‐fold of ULN (regardless of PPI use) or probable when FSG measurements were elevated consecutively (a) more than a two‐fold of ULN (without PPI) without consecutive FSG levels <2x ULN during follow‐up or (b) more than a five‐fold (under PPI) without consecutive FSG levels <5x ULN during follow‐up, without surgery or the start of systemic antitumor therapy (Table S1). Pathological gastrinoma diagnosis was established if immunohistochemistry of tumor tissue stained positive for gastrin, in the presence of hypergastrinemia.

### 2.3 Clinical definitions

The date of gastrinoma diagnosis was based on the date of the pathology report or on the date of the first FSG measurement fulfilling one of the diagnostic criteria. For nongastrinoma patients the date of the first FSG measurement was used. FSG levels at the time of gastrinoma diagnosis were regarded as initial FSG levels for further analysis.

Patients were categorized as a pathological or a biochemical diagnosis in line with whichever diagnosis came first.

Conventional imaging reports of computed tomography (CT), magnetic resonance imaging (MRI), endoscopic ultrasonography (EUS), or gastroduodenoscopy were reviewed for lesions suspicious for duodenal NETs and suspicious abdominal lymph nodes. Data from imaging reports up to 1 year before or after the gastrinoma diagnosis were extracted. Gastroduodenoscopies were also interpreted for gastric NETs. Lesions considered suspicious included visible tumor, polyposis, and small nodules. Gastritis and hypergastrinemia‐related complications were not considered as NET if there was no lesion suspicious for gastrinoma.

Liver metastases were defined as (a) pathologically proven or (b) radiologically confirmed liver metastases. Radiology was considered positive if consecutive CT or MRI reports described suspicious liver lesions. An MEN1 expert panel, blinded to patient identity, decided most likely origin of the liver metastases. The presence of synchronous liver metastases was studied as prognostic factor, regardless of origin.

Deaths caused by MEN1 manifestations and MEN1‐related therapy were considered as MEN1 related. Other causes of death were regarded as non‐MEN1 related.^2^

MEN1‐related NETs at the moment of gastrinoma diagnosis were diagnosed according to pathology reports.^2^ If no pathology reports were available, imaging results were used for diagnosis as previously described.^19^ Gastric NETs were also diagnosed using gastroduodenoscopies. The size of the largest pNET on conventional imaging was used for further analysis.

### 2.4 Treatment

Patients were treated medically by PPIs to prevent acid‐related complications by somatostatin analogs or surgically to prevent metastatic disease. Treatment regimen was decided by the treating physician together with the patient after multidisciplinary team discussion.

### 2.5 Outcome measures

The primary outcomes were 5‐ and 10‐year overall survival (OS). Possible prognostic factors at gastrinoma diagnosis were analyzed for influence on OS. The date of death or date of last follow‐up was used for analysis.

### 2.6 Statistical analysis

Descriptive statistics were reported as mean (standard deviation [SD]) or median (range), as appropriate, or as numbers (percentages). Differences in means were tested using t tests. Survival curves were plotted according to the Kaplan‐Meier method and survival probabilities were obtained.^20^ Follow‐up time started at the moment of gastrinoma diagnosis. Kaplan‐Meier curves were plotted for patients with MEN1 gastrinoma against nongastrinoma patients from the database. Concerning the difference age, patients with gastrinoma were 1:1 age and gender matched with a nongastrinoma patient. The log‐rank test was used for Kaplan‐Meier curve comparison.

Prognostic factors for OS were assessed using uni‐ and multivariable Cox proportional hazard regression providing hazard ratios (HR's) with 95% confidence intervals (95% CIs) ties were handled using the exact method. Cox proportional hazard assumptions were formally tested and graphically assessed using scaled Schoenfeld residual plots; the assumptions were not violated. Prognostic factors were adjusted for age, since age is associated with OS.^11^ Continuous variables were dichotomized based on previous literature; pNET size on conventional imaging to <2.0 cm and ≥2.0 cm, and FSG levels at gastrinoma diagnosis to <20x ULN and ≥20x ULN.^10^, ^11^

Since surgery might reduce FSG levels, subgroup analysis for the prognostic value of FSG levels was performed in nonsurgically managed patients. Furthermore, subgroup analysis was conducted in patients without liver metastases at gastrinoma diagnosis. All test were performed two‐tailed. *P*‐values <.05 were considered statistically significant. Statistical analysis was performed using SPSS version 25.0 (IBM Corp, New York), RStudio version 1.0.143 (RStudio, Inc., Boston, MA); figures were constructed using Graphpad Prism version 7.02 (GraphPad Software Inc, California).

## 3 RESULTS

### 3.1 DMSG database

A total of 396 patients were identified, of whom 357 (90%) had FSG measurements at least once between 1990 and 2014. The median number of measurements was 7 (1‐54) per patient. Hypergastrinemia was observed in 193 patients (54%), regardless of PPI use. In 114 patients (32%), FSG levels were >1x ULN in the absence of PPI. One hundred patients (28%) had FSG levels >2x ULN under PPI. Ten‐fold increased FSG levels were longitudinally observed in 45 patients (13%).

### 3.2 Patient characteristics

Demographic and clinical characteristics are described in Table 1. Sixty‐three patients with gastrinoma (16%) were identified in the DMSG database with a mean age of 51 years (±13). Fifty‐four percent were female. Most patients were diagnosed biochemically (64%) and 15 patients (24%) had a biochemical diagnosis with histopathological gastrinoma confirmation. In 22 patients (35%) gastrinomas were histopathologically proven. On the basis of biochemical criteria, 45 patients (71%) were diagnosed as certain and 10 (16%) as probable. Median FSG levels at diagnosis were 9.5x ULN (0.5‐412).

**Table 1:** Patient and disease characteristics at moment of gastrinoma diagnosis

	Overall patients (n = 63)
Age, mean [SD]	51 [13]
Gender	
Male (%)	29 (46%)
Female (%)	34 (54%)
MEN1‐associated tumors at the moment of gastrinoma diagnosis	
Pancreatic NET	33 (52%)
Gastric NET	7 (11%)
Lung NET	5 (8%)
Thymic NET	0
Gastrinoma diagnosis	
Pathological only	7 (11%)
Biochemical and pathological confirmation	15 (24%)
Biochemical only	40 (64%)
Imaging suspect for gastrinoma with elevated FSG levels	1 (2%)
Basis of biochemical gastrinoma diagnosis	
1 × >10x ULN	45 (71%)
2 × >2x ULN without PPI or >5x ULN with PPI	10 (16%)
FSG levels not fulfilling above criteria	8 (13%)
Fasting serum gastrin factor of ULN at diagnosis, median [range]^**^	
Overall (n = 61)	9.5 [0.5‐412.3]
No PPI, no somatostatin analogs (n = 21)	7.2 [1.4‐137.1]
Under PPI (n = 37)	9.64 [1.1‐412.3]
Under somatostatin analogs (n = 2)	45.7 [0.5‐90.9]
Under PPI and somatostatin analogs (n = 1)	19.2
Fasting serum gastrin factor of ULN at diagnosis, median [range]	
Biochemical diagnosis (n = 53)	11.0 [2.0‐412.3]
Pathological diagnosis (n = 7)	2.1 [0.5‐3.4]
Year of diagnosis	
Before 2007	31 (49%)
2007 and after	32 (51%)
Imaging suspicious for NET duodenum^***^	
Yes	15/5 (26%)
No	41/57 (74%)
Gastroduodenoscopy suspicious for NET duodenum^****^	
Yes	13/25 (52%)
No	12/25 (48%)
Patients with positive gastroduodenoscopy suspicious for NET duodenum	
Solitary lesion	5/13 (38%)
Multiple lesions	8/13 (62%)
Size duodenal abnormalities in mm, median [range] (n = 9)	7.5 [3‐20]
Gastroduodenoscopy suspicious for NET stomach	
Yes	7/25 (28%)
No	18/25 (72%)
Suspicious lymph nodes on imaging at gastrinoma diagnosis	
Yes	12 (19%)
No	51 (81%)
Liver metastases at diagnosis^*****^	5 (8%)
Gastrinoma	3
NF‐pNET	1
Gastrinoma or NF‐pNET	1

Abbreviations: FSG, fasting serum gastrin; NET, neuroendocrine tumor; NF‐pNET, nonfunctioning pancreatic neuroendocrine tumor; PPI, proton pump inhibitor; SD, standard deviation; ULN, upper limit of normal of the reference value.

*According to which diagnosis came first.

^**^ FSG levels are reported for subgroups on medical therapy (PPI, somatostatin analogs or both) at the moment of FSG measurement.

^***^ Imaging suspect for gastrinoma duodenum: abnormalities on computed tomography (CT), magnetic resonance imaging (MRI), gastroduodenoscopy, or endoscopic ultrasonography.

^****^ Gastroduodenoscopy suspicious for NET: visible tumor, polyposis without another diagnosis and small nodules which could be biopsied. Possible Zollinger‐Ellison syndrome‐related complications such as peptic ulcera were not considered as suspected for NET. Gastritis was not documented as suspect for NET.

^*****^ Origin of liver metastases according to the expert panel.

Thirty‐five patients (56%) harbored a concurrent NET at the time of gastrinoma diagnosis. Thirty‐three patients had a concurrent pNET (52%). Eight patients had a pNET and concurrent lung or gastric NET. One patient with a pathologically confirmed gastric gastrinoma also had a concurrent gastric NET. Five patients (8%) harbored synchronous liver metastases. For two patients the liver metastases were pathologically proven; nonfunctioning pNET (NF‐pNET) liver metastases in one and gastrinoma‐related in another.

Forty‐seven patients (75%) received medical treatment and 16 patients (25%) underwent surgery (Figure S1). Two patients additionally received peptide receptor radionuclide therapy, of whom one died during the follow‐up.

### 3.3 Long‐term outcomes

Patient outcomes are reported in Table 2. Eight patients (14%) developed liver metastases after a median follow‐up of 4.5 years (0.3–23.5 years). Liver metastases of any MEN1‐related manifestation were confirmed by pathology in three patients, whereas in five patients the diagnosis and origin were established by the expert panel. Gastrinoma liver metastases were pathologically confirmed in one patient. After a follow‐up of 4.7 years, 17 patients (27%) had died; at a median age of 58 years (33–81 years). Eleven deaths (65%) were regarded as MEN1 related. Most MEN1‐related deaths (73%) resulted from dpNET progression. Two patients were lost during the follow‐up.

**Table 2:** Survival and long‐term outcomes of patients with MEN1 gastrinoma

	Overall patients (n = 63)
Follow‐up in years, median [range]*	4.7 [0.25‐23.5]
Overall survival	
5‐y, % (95% CI)	83% (68‐92%)
10‐y, % (95% CI)	65% (47‐79%)
Liver metastases^**^	8 (14%)
Gastrinoma	3
NF‐pNET	2
Gastrinoma or NF‐pNET	1
Thymic NET	1
Unknown origin/unknown if MEN1 dpNET related	1
Death	17 (27%)
MEN1‐related	11 (65%)
Duodenopancreatic NET related	8
Thymic NET	1
Renal insufficiency caused by pHPT	1
Complication MEN1 pancreatic surgery	1
Non‐MEN1‐related	5 (29%)
Unknown	1 (6%)

Abbreviations: dpNET, duodenopancreatic neuroendocrine tumor; MEN1, multiple endocrine neoplasia type 1; NF‐pNET, nonfunctioning pancreatic neuroendocrine tumor; NET, neuroendocrine tumor; pHPT, primary hyperparathyroidism.

* Follow‐up until death or end of follow‐up.

^**^ Origin of liver metastases is based on the expert panel. Percentage is based on the group of patients without liver metastases at diagnosis (n = 58).

### 3.4 Survival of patients with MEN1 gastrinoma

**Figure 1:**
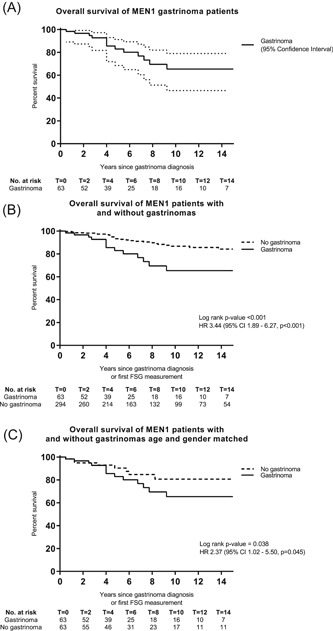
Overall survival (OS) of MEN1 gastrinoma patients (A). OS of MEN1 patients with and without gastrinomas (B). OS of MEN1 patients with and without gastrinomas (age and gender matched) (C). MEN1, multiple endocrine neoplasia type 1

OS rates of patients with MEN1 gastrinoma after 5 and 10 years were 83% and 65%, respectively (Table 2; Figures 1A and S2A). Five and 10‐year OS rates for patients with MEN1 having FSG measurements not indicative for a gastrinoma were 93% and 87%, respectively (Figures 1B and S2B). Patient with gastrinomas were older than patients without gastrinomas (51 vs 39 years, *P* < .001). Ten‐year OS rates were 65% vs 81% for age and gender matched MEN1 patients without gastrinomas (Figures 1C and S2C).

### 3.5 Prognostic factors for OS

Prognostic factors for OS are shown in Table 3. Factors significantly associated with OS were FSG levels >20x ULN (HR, 6.16; [95% CI, 1.65‐23.02]), pNET ≥2.0 cm on conventional imaging (HR, 4.46; [1.52‐13.06]), synchronous liver metastases of any origin (HR, 8.86; [2.14‐36.7]), multiple concurrent NETs (HR, 5.86; [1.24‐27.65]), and gastroduodenoscopy suspicious for gastric NET (HR, 12.74; [1.40‐115.6]). After adjusting for age, these factors were significantly associated with OS.

**Table 3:** Cox proportional hazards regression for prognostic factors for overall survival

	N	Deaths	Overall survival probabilities		Unadjusted HR (95% CI), *P*‐value	Age‐adjusted HR (95% CI), *P*‐value
			5‐y	10‐y		
Age	63	17	NA	NA	1.04 (0.99‐1.09), *P* = 0.116	NA
Gender						
Male	29	8	81%	60%	1 (Ref. cat.)	1 (Ref. cat.)
Female	34	9	85%	71%	0.60 (0.21‐1.70), *P* = .335	0.55 (0.19‐1.62), *P* = .283
MEN1‐associated NETs at gastrinoma diagnosis						
No concurrent NET	27	6	94%	72%	1 (Ref. cat.)	1 (Ref. cat.)
Pancreatic NET	25	7	79%	69%	1.46 (0.49‐4.38), *P* = .499	1.93 (0.61‐6.13), *P* = .263
Gastric NET	3	1	67%	67%	2.38 (0.27‐20.83), *P* = .433	7.20 (0.67‐77.46), *P* = .103
Multiple (pNET, gastric NET, and/or lung NET)	8	3	56%	0%	5.86 (1.24‐27.65), *P* = .026	9.73 (1.77‐53.44), *P* = .009
Initial gastrinoma diagnosis						
Histopathological diagnosis	9	1	89%	89%	1 (Ref. cat.)	1 (Ref. cat.)
Biochemical diagnosis	53	16	82%	63%	1.54 (0.20‐11.99), *P* = .68	1.04 (0.12‐8.50), *P* = .971
Basis of gastrinoma diagnosis						
No 1 × >10x ULN	18	1	94%	94%	1 (Ref. cat.)	1 (Ref. cat.)
1 × >10x ULN	45	16	80%	61%	3.09 (0.40‐24.03), *P* = .282	3.11 (0.40‐24.31), *P* = .280
Basis of gastrinoma diagnosis						
No 2 × >2x ULN without PPI or >5x ULN with PPI	53	16	83%	64%	1 (Ref. cat.)	1 (Ref. cat.)
2 × >2x ULN without PPI or >5x ULN with PPI	10	1	88%	88%	0.67 (0.09‐5.21), *P* = .701	0.62 (0.08‐4.80), *P* = .645
Fasting serum gastrin levels at diagnosis						
<10x ULN	33	3	91%	84%	1 (Ref. cat.)	1 (Ref. cat.)
≥10x ULN & <20x ULN	13	4	88%	66%	2.66 (0.57‐12.27), *P* = .214	2.40 (0.51‐11.33), *P* = .266
≥20x ULN	15	10	65%	33%	6.16 (1.65‐23.02), *P* = .007	5.56 (1.45‐21.27), *P* = .012
Conventional imaging suspicious for NET duodenum*						
No	42	9	85%	69%	1 (Ref. cat.)	1 (Ref. cat.)
Yes	15	5	79%	26%	2.82 (0.87‐9.15), *P* = .084	2.81 (0.86‐9.13), *P* = .087
Gastroduodenoscopy suspicious for NET duodenum						
No	12	2	89%	71%	1 (Ref. cat.)	1 (Ref. cat.)
Yes	13	4	85%	28%	2.11 (0.38‐11.62), *P* = .391	2.61 (0.46‐14.87), *P* = .281
Gastroduodenoscopy suspicious for NET stomach						
No	18	2	100%	69%	1 (Ref. cat.)	1 (Ref. cat.)
Yes	7	4	57%	0%	12.74 (1.40‐116), *P* = .024	18.97 (1.62‐222), *P* = .019
Pancreatic NET at gastrinoma diagnosis						
No	30	7	92%	72%	1 (Ref. cat.)	1 (Ref. cat.)
Yes	33	10	74%	59%	1.70 (0.64‐4.50), *P* = .288	1.97 (0.71‐5.45), *P* = .190
Pancreatic NET ≥ 2.0 cm on imaging at gastrinoma diagnosis						
No	51	11	89%	71%	1 (Ref. cat.)	1 (Ref. cat.)
Yes	12	6	56%	42%	4.46 (1.52‐13.06), *P* = .006	6.71 (2.02‐22.4), *P* = .002
Suspicious lymph nodes on imaging at gastrinoma diagnosis						
No	51	15	80%	64%	1 (Ref. cat.)	1 (Ref. cat.)
Yes	12	2	100%	67%	0.88 (0.20‐3.95), *P* = .868	1.16 (0.24‐5.52), *P* = .852
Liver metastases at gastrinoma diagnosis						
No	58	14	88%	69%	1 (Ref. cat.)	1 (Ref. cat.)
Yes	5	3	25%	‐	8.86 (2.14‐36.7), *P* = .003	6.56 (1.42‐30.38), *P* = .016

Abbreviations: HR, hazard ratio; NA, not applicable; NET, neuroendocrine tumor; PPI, proton pump inhibitor; ULN, upper limit of the normal of the reference value.

* Conventional imaging: magnetic resonance imaging, computed tomography, endoscopic ultrasonography, or gastroduodenoscopy suspicious for duodenal gastrinoma. In one case the gastroduodenoscopy was suspicious for a gastric gastrinoma.

**Figure 2:**
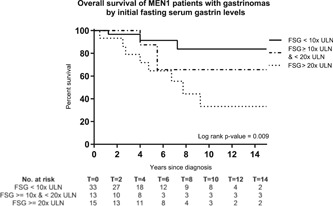
Overall survival of patients with MEN1 according to initial fasting serum gastrin levels. MEN1, multiple endocrine neoplasia type 1

Initial FSG levels determined prognosis in patients with MEN1 gastrinoma. Ten year OS was more favorable for patients with FSG <10x ULN compared with patients with FSG ranging from 10 to 20x ULN and FSG ≥20x ULN (83%, 66%, and 33%, respectively) (Figures 2 and S3). Corresponding HR's were 2.66 ([0.57‐12.27]; *P* = .214) for FSG ranging from 10 to 20x ULN and 6.16 ([1.65‐23.02]; *P* = .007) for FSG ≥20x ULN. Comparable results were observed in nonsurgically managed patients and patients without synchronous liver metastases (Table 4).

**Table 4:** Prognostic value of initial fasting gastrin levels on overall survival (OS)

	Overall cohort (n = 63)		Nonsurgically managed patients (n = 47)		Patients without liver metastases (n = 58)	
	10‐y OS	HR (95% CI), *P*‐value	10‐y OS	HR (95% CI), *P*‐value	10‐y OS	HR (95% CI), *P*‐value
Fasting serum gastrin levels						
<10x ULN	84%	1 (Ref. cat.)	88%	1 (Ref. cat.)	92%	1 (Ref. cat.)
≥10x ULN & <20x ULN	66%	2.66 (0.57‐12.27), *P* = .214	53%	3.67 (0.58‐23.07), *P* = .165	66%	7.53 (0.82‐69.46), *P* = .075
≥20x ULN	33%	6.16 (1.65‐23.02), *P* = .007	25%	8.40 (1.76‐40.03), *P* = .008	33%	17.34 (2.15‐140.21), *P* = .007

Abbreviations: CI, confidence interval; HR, hazard ratio; OS, overall survival; ULN, upper limit of the normal of the reference value.

## 4 DISCUSSION

Gastrinomas in patients with MEN1 lead to a decreased life expectancy with 5 and 10‐year OS rates of 83% and 65%, respectively. Factors associated with decreased OS were initial FSG levels ≥20x ULN, a pNET ≥2.0 cm on conventional imaging, synchronous liver metastases, multiple concurrent NETs, and gastroduodenoscopy suspicious for gastric NET. These factors may guide clinical decision making in daily practice.

The 10‐year OS rate in our cohort of patients with gastrinoma was 65% with a median age at death of 58 years. Overall, patients with MEN1 in the Netherlands have a life expectancy of 73 years.^2^ We performed subgroup analysis in age and gender matched controls, also showing a significantly decreased OS of MEN1 gastrinomas. This underscores that age and gender did not influence this outcome. Previous studies on patients with MEN1 gastrinoma reported 10‐year survival rates of 88% to 100% regardless of therapy.^7^, ^8^, ^11^, ^12^, ^13^ Several factors could account for the different survival rates. First, disease‐specific survival is generally higher than OS.^10^, ^11^, ^12^ Second, in the other cohorts also nongastrinoma patients might have been included because of the method of identifying gastrinomas. Finally, patient prognosis might be influenced by treatment regimen. In our cohort, only 25% of the patients underwent surgery and in a substantial part the duodenum was not removed, which is deemed necessary for achieving biochemical cure.^8^ In addition, patients with synchronous liver metastases were included in our study. Liver metastases, either NF‐pNET or gastrinoma related, are associated with survival.^7^, ^11^, ^13^, ^17^, ^21^ Although the OS rate was lower, the age of death (58 years) was slightly higher than in previous series (55‐56 years).^10^, ^11^, ^12^

Initial FSG levels were associated OS, also after adjusting for age. More specifically, OS decreased as FSG levels increased. Ito et al^10^ described very high FSG levels (>20‐fold elevated) more often in deceased patients. We observed a significantly increased HR for death in patients with FSG levels higher than 20x ULN. In line, we observed a HR of 2.66 and a 10‐year OS of 66% for patients with FSG levels between 10 and 20x ULN. We believe that the outcomes of this analysis were not statistically significant due to the low number of patients and events in this subgroup. NIH series observed higher FSG levels in patients with an aggressive disease course and in patients with liver metastases, although no survival analysis was conducted.^7^, ^13^ The only study focusing on initial FSG levels and survival in patients with MEN1 from the NIH, did not find a significant correlation in MEN1 gastrinoma patients (log rank *P* = .068).^22^ In this study of 53 patients with MEN1 gastrinoma, only four deaths were observed.^22^ Compared with our study, in this NIH series other diagnostic criteria were applied probably influencing the case mix and, in addition, the study period was a decade earlier.

Pancreatic NET ≥2.0 cm on imaging and liver metastases at gastrinoma diagnosis were associated with decreased OS. Formerly, gastrinomas were generally regarded as pNETs causing liver metastases and death. However, studies including gastrin immunohistochemistry and pathological series report the predominant duodenal origin.^5^, ^8^, ^23^ Therefore, it can be hypothesized that these patients have decreased OS because of a concurrent NF‐pNET ≥2.0 cm instead of a pancreatic gastrinoma. Recently, the acceptable prognosis of NF‐pNETs <2 cm has been highlighted.^19^, ^24^, ^25^ The decreased survival of patients with MEN1 having liver metastases is in line with other studies.^11^, ^13^ Nevertheless, the exact cause of death in patients with MEN1 gastrinoma having concurrent large (NF)‐pNETs remains challenging.

This study is limited by the challenges of gastrinoma diagnosis in patients with MEN1. Current guidelines recommend the combination of hypergastrinemia and basal gastric acid hypersecretion (pH < 2).^14^, ^15^ Gastrinoma diagnosis frequently differs from these criteria, because of the lack of gastric pH measurement, the unavailability of secretin testing and the use of immunohistochemistry and imaging as alternatives.^26^ In daily clinical practice of the DMSG, gastrinoma diagnosis was complicated by the unavailability of stimulation tests, no routine measurements of gastric pH and widespread use of PPI. Because we aimed to study OS and predictors of OS, we wanted to be sure to select gastrinoma patients only. Therefore, strict selection criteria were formulated beforehand. Only 11% did not have 10‐fold increased FSG levels nor pathologically proven gastrinoma. To identify this subgroup of gastrinoma cases, we reasoned that gastrinomas lead to gradual FSG increases, therefore, longitudinally collected FSG values were analyzed and patients with spontaneously decreasing values over time were not regarded as patients with gastrinoma. FSG measurements in light of the annual MEN1 screening were performed regardless of PPI use. Although PPIs are preferably discontinued before FSG measurement, serious adverse events can occur during sudden interruption.^27^ Thus, we believe that for identifying patients with MEN1 gastrinoma including serial FSG measurements provide a more pragmatic, but still reliable approach. Other limitations include the retrospective design and the number of events. Due to the low number of events, extensive multivariable analysis was impossible and relatively wide confidence intervals were observed.

The major strength of this study is the population‐based cohort including >90% of patients with MEN1, with standardized data collection and long‐term follow‐up. In addition, patients are included from 1990 onwards, providing more actual survival rates, since patients with MEN1 are a biochemically screened population and gastric acid hypersecretion‐related deaths have been rarely reported over the last two decades.^9^, ^10^ In the present study, OS was used as outcome, because OS is more informative and establishing gastrinoma‐related deaths (disease‐specific survival) is challenging in the presence of multiple MEN1 manifestations. Furthermore, this is the first study to assess prognostic factors, including FSG levels, on OS in patients with MEN1 using time‐to‐event analysis.

The observed prognostic factors might aid clinicians in selecting patients with MEN1 having gastrinomas for more intensive follow‐up regimens or extended localization imaging. Furthermore, knowledge of prognostic factors and survival can help in selecting those who might benefit from surgery. MEN1 and ENETS guidelines recommend surgery for patients with MEN1 having pancreatic gastrinomas >2.0 cm.^14^, ^15^ Although 52% had a pNET at the moment of gastrinoma diagnosis, only 19% of all patients in this cohort had pNETs >2.0 cm on cross‐sectional imaging. Especially, in the coexistence of hypergastrinemia and pNETs ≥2.0 cm, the optimal surgical strategy is hard to establish. Merging the need for pancreaticoduodenal resections to achieve biochemical cure on the one hand and the scarcity of data regarding postoperative complications and long‐term oncological outcomes after pancreaticoduodenal resections on the other, future studies should address these topics to come to meaningful advice.^28^

In conclusion, life expectancy in patients with MEN1 having gastrinomas is reduced compared with other studies. OS was associated with initial FSG levels ≥20x ULN, a pNET ≥2.0 cm on conventional imaging, synchronous liver metastases, multiple concurrent NETs, and gastroduodenoscopy suspicious for gastric NET in patients with MEN1. OS decreases as FSG levels increase, starting from ≥10x ULN. Therefore, FSG levels might provide a valuable tool to guide surveillance intensity, step‐up to additional diagnostic modalities or provide arguments in selecting those patients who might benefit from surgery.

## FUNDING

This study was supported by an unrestricted grant from Ipsen Pharmaceutical. The funding source had no influence on the study question, design, data acquisition, statistical analysis, and interpretation of data.

## CONFLICT OF INTERESTS

There is no conflict of interest that could be perceived as prejudicing the impartiality of the research reported.

## AUTHOR CONTRIBUTIONS

DJvB: study design, statistical analysis and interpretation of data, drafting, and final approval of the manuscript.

SN: study design, acquisition of data, interpretation of data, critical revision, and final approval of the manuscript.

CRCP: study design, design of data collection protocol, acquisition of data, critical revision, and final approval of the manuscript.

WWdH: study design, critical revision, and final approval of the manuscript.

ACvdV: study design, critical revision, and final approval of the manuscript.

OMD: study design, critical revision, and final approval of the manuscript.

ANvdH‐S: study design, critical revision, and final approval of the manuscript.

MLD: study design, critical revision, and final approval of the manuscript.

PHB: study design, critical revision, and final approval of the manuscript.

BH: study design, critical revision, and final approval of the manuscript.

IHMBR: study design, critical revision, and final approval of the manuscript.

MRV: study design, interpretation of data, critical revision, and final approval of the manuscript and study supervision.

GDV: study design, interpretation of data, critical revision, and final approval of the manuscript and study supervision.

## DATA AVAILABILITY

The data that support the findings of this study are available on request from the corresponding author. The data are not publicly available due to privacy or ethical restrictions.

## DISCLOSURE SUMMARY

The authors have nothing to disclose.

## SYNOPSIS

Prognostic factors of survival in multiple endocrine neoplasia type 1 (MEN1) gastrinomas were studied. Life expectancy of MEN1 gastrinoma patients is reduced. Fasting serum gastrin levels and pancreatic neuroendocrine tumors ≥2 cm are prognostic factors.

## Supporting information


**Supplementary Figure 1:** Flow‐chart of MEN1‐related gastrinomas treatment

**Supplementary Figure 2 (color version):** Overall survival (OS) of MEN1 gastrinoma patients (A). OS of MEN1 patients with and without gastrinomas (B). OS of MEN1 patients with and without gastrinomas (age and gender matched) (C)

**Supplementary Figure 3 (color version):** Overall survival of MEN1 patients according to initial fasting serum gastrin levels

Supporting information
