# Supplementary figures and images for: Prognostic factors and survival in MEN1 patients with gastrinomas: Results from the DutchMEN study group (DMSG)
Source: J Surg Oncol. 2019 Aug 10;120(6):966–75. doi: 10.1002/jso.25667 (PMC6852496; doi:10.1002/jso.25667)

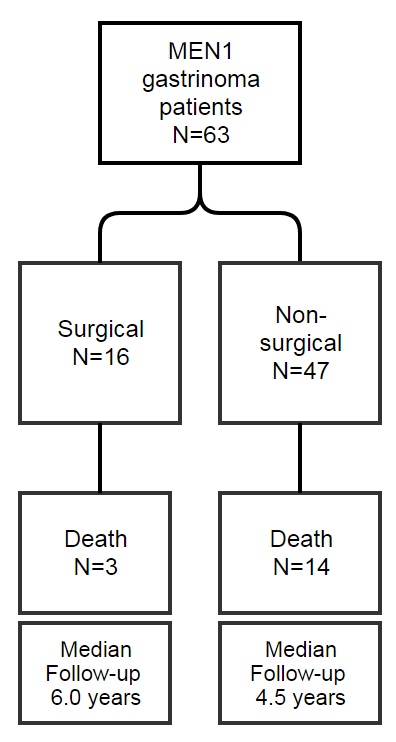

Supplement: Supplementary file 1 — Supplementary Figure 1: Flow‐chart of MEN1‐related gastrinomas treatment [file JSO-120-966-s001.jpg]
