# Supplementary material for: Prognostic factors and survival in MEN1 patients with gastrinomas: Results from the DutchMEN study group (DMSG)
Source: J Surg Oncol. 2019 Aug 10;120(6):966–75. doi: 10.1002/jso.25667 (PMC6852496; doi:10.1002/jso.25667)
Supplement: Supplementary file 4 — Supporting information [file JSO-120-966-s004.docx]

| **Supplementary table 1. Biochemical gastrinoma diagnosis DutchMEN Study Group database**^19^**.** | |
| --- | --- |
| Gastrinoma  Certain | FSG >10 times the upper limit of normal (ULN) |
| Gastrinoma  Probable | FSG >2 times twice consecutive in the absence of proton pump inhibitor use (no value <2 ULN allowed in between) and not followed by two consecutive measurements <2 ULN without surgery or start of systemic anti-tumor therapy |
|  | FSG >5 times twice consecutive in the presence of proton pump inhibitor use (no value <5 ULN allowed in between) and not followed by two consecutive measurements <5 ULN without surgery or start of systemic anti-tumor therapy |
| Abbreviations: *FSG* Fasting serum gastrin, *ULN* Upper limit of normal. | |
